# Computer Simulation of Sulfated Cyclodextrin–Based Enantioselective Separation of Weak Bases With Partial, High‐Concentration Filling of the Chiral Selector and Analyte Detection on the Cathodic Side

**DOI:** 10.1002/elps.202400213

**Published:** 2025-01-12

**Authors:** Friederike A. Sandbaumhüter, Wolfgang Thormann

**Affiliations:** ^1^ Medical Mass Spectrometry, Department of Pharmaceutical Biosciences, Biomedical Centre 591 Uppsala University Uppsala Sweden; ^2^ Institute for Infectious Diseases University of Bern Bern Switzerland

**Keywords:** capillary electrophoresis, capillary electrophoresis–mass spectrometry (CE–MS), chiral separation, computer simulation, sulfated cyclodextrin

## Abstract

Computer simulation was utilized to characterize the electrophoretic processes occurring during the enantioselective capillary electrophoresis–mass spectrometry (CE–MS) analysis of ketamine, norketamine, and hydroxynorketamine in a system with partial filling of the capillary with 19 mM (equals 5%) of highly sulfated γ‐cyclodextrin (HS‐γ‐CD) and analyte detection on the cathodic side. Provided that the sample is applied without or with a small amount of the chiral selector, analytes become quickly focused and separated in the thereby formed HS‐γ‐CD gradient at the cathodic end of the sample compartment. This gradient broadens with time, remains stationary, and gradually reduces its span from the lower side due to diffusion such that analytes with high affinity to the anionic selector become released onto the other side of the focusing gradient where anionic migration and defocusing occur concomitantly. The analytes that remain focused until the migrating HS‐γ‐CD concentration boundary arrives at the cathodic end of the sample compartment become gradually released into the cathodic part and migrate in the absence of HS‐γ‐CD toward the detector. This behavior is dependent on the length of the HS‐γ‐CD zone in the cathodic part of the electrophoretic column, the initial sample zone length, and the sample matrix. The data presented reveal the possibility that only one of the enantiomers of an analyte migrates toward the detector, whereas the other is lost for the analysis, or that both enantiomers migrate toward the cathode but do not separate. Enantiomer separation followed by migration toward the cathode can only be achieved for analytes with rather low complexation constants, such as hydroxynorketamine assessed in this work, and is dependent on the slope of the HS‐γ‐CD focusing gradient. The gained insights illustrate that dynamic simulation is an indispensable tool to investigate electrophoretic processes of complex systems.

**Abbreviations**
HS‐γ‐CD: highly sulfated γ‐cyclodextrin
NMDA receptor: *N*‐methyl‐d‐aspartate receptor

## 1 Introduction

Enantioselective analysis of chiral drugs and their metabolites provides insights into their pharmacological activity, mechanism of action, as well as safety and efficacy profiles. Furthermore, governmental regulations require to monitor chirality under drug development. Chirality can affect pharmacokinetics and pharmacodynamics, leading to differences in metabolism, distribution, and potential side effects of enantiomers [1, 2, 3]. Ketamine is a chiral drug with anesthetic, analgesic, and antidepressive properties. Both S‐ and R‐ketamine are pharmacologically active, but S‐ketamine has a higher affinity to the *N*‐methyl‐d‐aspartate receptor and provides a stronger inhibition. The metabolism of ketamine is enantioselective, and the metabolites norketamine and hydroxynorketamine are pharmacologically active themselves [4, 5, 6, 7, 8, 9]. Thus, enantioselective analysis of ketamine and its metabolites is crucial for understanding the mechanism of action and a safe and efficient application in patients. Enantioselective assays based on capillary electrophoresis (CE) with sulfated cyclodextrin (CD) as a chiral selector have been widely used to study the metabolism and pharmacokinetics of ketamine and its metabolites [10, 11, 12, 13, 14, 15, 16, 17].

CE plays a fundamental role in chiral analysis due to its high enantioselective separation efficiency and compatibility with different detection techniques [18, 19]. Additionally, only a few nanoliters of sample are required, and low solvent consumption makes it a cost‐effective and environmentally friendly method in biomedical analysis [2, 3, 20, 21, 22, 23]. The integration of CE with mass spectrometry (MS) is advantageous for a precise and accurate analysis with high sensitivity and selectivity of chiral drugs, metabolites, and biomarkers [3, 24, 25, 26]. When coupling CE to MS, the CE separation conditions must be compatible with MS detection, and the interface between CE and MS must be carefully optimized to ensure efficient ionization. In all CE techniques, enantiomer separation is based on the formation of diastereomeric complexes that have different effective mobilities and occurs while analytes are exposed to a chiral selector. To reduce the amount of chiral selector that reaches the MS detector and to consequently minimize ion suppression and background noise, the partial filling technique is often applied, which comprises the strategic filling of only a part of the separation capillary with the chiral selector [17, 18, 25, 27, 28, 29].

**FIGURE 1:**
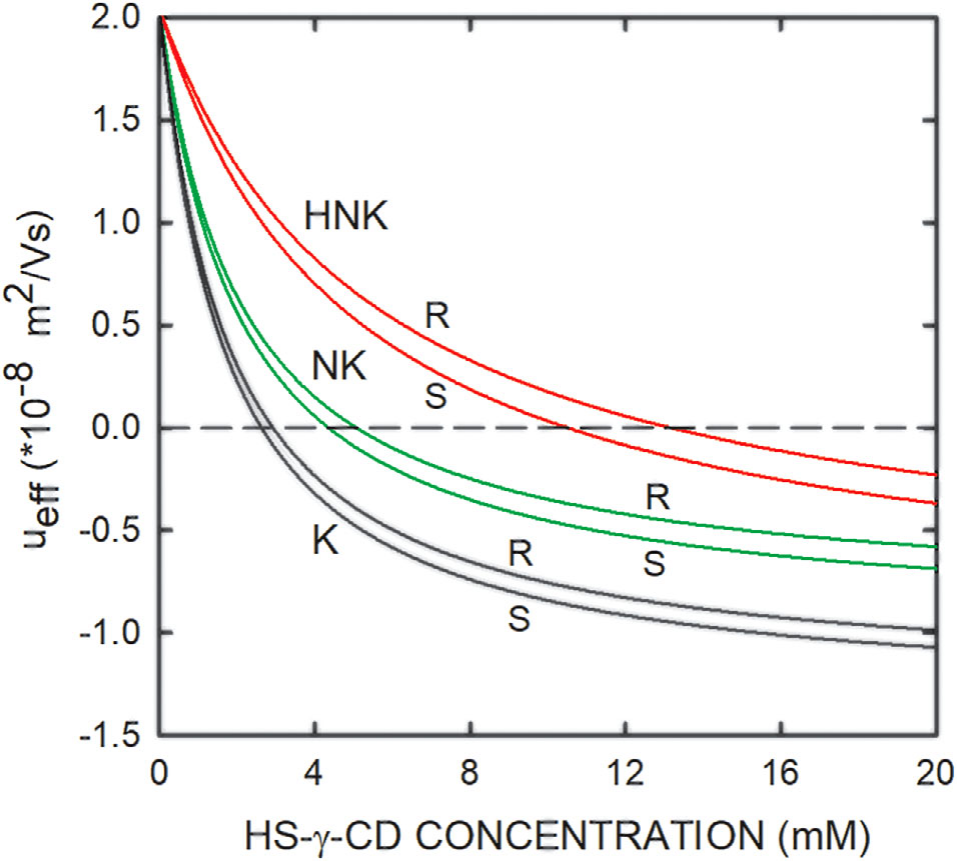
Effective mobility versus HS‐γ‐CD concentration for S‐ and R‐ketamine (black line graphs), S‐ and R‐norketamine (green line graphs), and S‐ and R‐hydroxynorketamine (red line graphs). K, NK, and HNK refer to ketamine, norketamine, and hydroxynorketamine, respectively. For details, refer to text. HS‐γ‐CD, highly sulfated γ‐cyclodextrin.

In the chiral CE‐based assays for ketamine, norketamine, and hydroxynorketamine, sulfated CDs were used as chiral selectors [10, 11, 12, 13, 14, 15, 16, 17]. These selectors are multiple isomer mixtures with multiple negative charges [30]. Ketamine, norketamine, and hydroxynorketamine are weak bases. At low pH, complexation of these bases with sulfated CDs results in a migration direction that is dependent on the concentration of the chiral selector. The migration direction is cationic at low CD concentration, becomes zero at a characteristic CD concentration, and is anionic for conditions with a higher CD concentration (Figure 1). The CE–MS system that was developed to selectively analyze stereoisomers of hydroxynorketamine in urine is based on partial filling with a BGE containing a high concentration (5% solution that equals 19 mM) of highly sulfated γ‐CD (HS‐γ‐CD) and featuring analyte detection at the cathodic capillary end [17]. At this CD concentration, ketamine, norketamine, and hydroxynorketamine stereoisomers migrate anionically toward the anode (Figure 1). Thus, how was it possible to monitor the hydroxynorketamine stereoisomers without buffer flow toward the cathode? A detailed answer to this question is the subject of this article. The electrophoretic processes occurring in this configuration with partial filling were analyzed by dynamic computer simulation, an approach that provides insight into electrokinetic separations, including those based on differential chiral interactions [31, 32, 33, 34, 35, 36].

In this work, the dynamic electrophoresis simulator SIMUL5complex [31, 32] that includes interactions of analytes with multiply charged ligands was used to gain insight into the electrokinetic processes leading to the chiral separation of S‐ and R‐ketamine and its metabolites S‐ and R‐norketamine and S‐ and R‐hydroxynorketamine in the CE–MS assay reported previously [17]. It is important to note that hydroxylation of norketamine occurs at different positions on the cyclohexanone and the chlorophenyl ring [11, 12]. To simplify matters, only one pair of hydroxynorketamine stereoisomers (referred to as S‐hydroxynorketamine and R‐hydroxynorketamine) was considered in the simulation work. Simulation was previously employed to characterize various preparations of sulfated β‐CDs [37] and to study the migration of ketamine and norketamine enantiomers in the presence of HS‐γ‐CD in an on‐line CE‐based assay format [38]. For the investigated CE–MS system, simulation is shown to provide insight into the enantiomer separation mechanism that includes the focusing of the analytes in a stationary gradient of the chiral selector prior to the cationic migration during the time interval of decreasing and vanishing concentration of the chiral selector. Focusing of analytes in a chiral selector gradient is described here for the first time. Furthermore, the impact of the length and composition of the sample zone as well as the length of the chiral selector zone and the slope of the focusing gradient, on the separation and migration of ketamine, norketamine, and hydroxynorketamine enantiomers was elucidated and compared to the experimental results reported previously [17]. The obtained data support future method development and optimization of the CE–MS assay.

## 2 Material and Methods

All simulations were performed with the software SIMUL5complex that can be downloaded as freeware from http://echmet.natur.cuni.cz/download. It is a 1D dynamic electrophoresis simulator that includes algorithms to describe 1:1 equilibria between solutes and a buffer additive such as a chiral selector [31, 32]. Apparent complexation constants, *K*, between the interacting species and the chiral selector and mobilities of the formed complexes, *u*_c_, are required as input. For ketamine and norketamine, these parameters were taken from a previous publication in which they were determined experimentally at conditions of different ionic strength [38], whereas those for hydroxynorketamine were estimated on the basis of chiral CE data. It is important to note that SIMUL5complex does not consider any corrections for ionic strength or viscosity. All buffer constituents were assumed not to interact with HS‐γ‐CD. The input data for all components used in the simulations are given in Table 1. The multiple isomer mixture HS‐γ‐CD was described as a single isomer assuming a degree of substitution and negative charge of 13 [38]. The effective mobilities of the six analytes as a function of the concentration of the chiral selector HS‐γ‐CD are presented in Figure 1.

**TABLE 1:** Input parameters used for simulations.

Component	Mobility 10^−8^ [m^2^/Vs]	p*K* _a_	*K* [M^−1^]	Complex mobility 10^−8^ [m^2^/Vs]
Formic acid	5.66	3.75	—	—
Acetic acid	4.42	4.76	—	—
Sodium	5.19	13.70	—	—
Chloride	7.91	−2.00	—	—
NH_3_	7.62	9.25	—	—
HS‐γ‐CD	2.00	−3.00^d^	—	—
S‐Ketamine	2.50	7.50^c^	579.0^a^	−1.34^a^
R‐Ketamine	2.50	7.50^c^	554.3^a^	−1.26^a^
S‐Norketamine	2.50	6.65^c^	482.7^a^	−0.97^a^
R‐Norketamine	2.50	6.65^c^	467.6^a^	−0.86^a^
S‐Hydroxynorketamine	2.50	6.65^b^	200.0^b^	−0.97^b^
R‐Hydroxynorketamine	2.50	6.65^b^	180.0^b^	−0.86^b^

^a^ Experimentally determined complexation values that were used as inputs for charged and neutral species of ketamine and norketamine [38].

^b^ Estimated values.

^c^ Input values from [38].

^d^ Value for each sulfate group.

**FIGURE 2:**
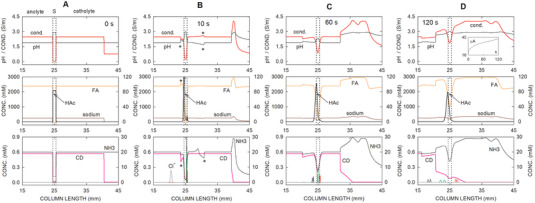
Computer‐predicted dynamics of an arrangement with partial filling of the chiral selector containing catholyte (15 mm zone) for (A) 0 s, (B) 10 s, (C) 60 s, and (D) 120 s after application of the separation voltage of 400 V. Bottom panels: Distributions of HS‐γ‐CD (pink line graph) and NH_3_ with the *y*‐axis on the right, and profiles of ketamine (black line graphs), norketamine (green line graphs), and hydroxynorketamine (red line graphs) with the *y*‐axis on the left. Center panels: Concentrations of formic acid (orange line) with the *y*‐axis on the left, and sodium (brown line) and acetic acid (black line) with the *y*‐axis on the right. Top panels: Distributions of pH (black line) and conductivity (red line). The cathode is on the right. The inset in the top panel of (D) presents the current as a function of time. The dotted vertical lines demarcate the boundaries of the sampling compartment. The asterisks in (B) mark the positions of the migrating system peaks. FA, HAc, CD, and S refer to formic acid, acetic acid, the chiral selector HS‐γ‐CD, and the sample compartment, respectively.

If not stated otherwise, the BGE with the chiral selector was composed of 2.36 M formic acid, 20 mM ammonium formate, 19 mM HS‐γ‐CD, and 247 mM sodium hydroxide (calculated pH of 1.90), and the catholyte on the detection side contained the same buffer without the chiral selector [17]. The sample consisted of stereoisomer mixtures of ketamine, norketamine, and hydroxynorketamine (20 µM of each stereoisomer as hydrochloride in 83 mM acetic acid). The electrophoretic column length used for simulation was 50 mm, and the sample‐BGE boundary widths were 0.05 mm. The configuration assessed comprises the sample in the column center (between 24.5 and 25.5 mm of column length) bracketed by BGE, as is shown in Panel A of Figure 2. Simulations were run with 20 000 segments (Δ*x* = 5 µm), under a constant voltage of 400 V and without the application of buffer flow (absence of electroosmosis). With a maximum error setting of 10^−5^, simulations took about 1 week to complete 3 min of simulated electrophoresis time using a Windows 10–based PC in the 64 bit format featuring an Intel Core I7‐10700K processor (8 cores, 16 threads) running at 3.8 GHz and 32 GB RAM. For making plots, data were imported into the SigmaPlot Scientific Graphing Software Windows Version 12.5 (SPSS, Chicago, IL, USA).

## 3 Results and Discussion

### 3.1 The Dynamics of the System With Partial Filling of the Negatively Charged Chiral Selector

The simulation data presented in Figure 2 depict the dynamics of the CE‐MS system with ketamine, norketamine, and hydroxynorketamine enantiomers as analytes, the BGE, and sample matrix compounds formic acid, ammonia, HS‐γ‐CD, sodium, and acetic acid, as well as a 15 mm long zone of HS‐γ‐CD on the cathodic side of the sample. The HS‐γ‐CD concentration is 19 mM, which corresponds to a 5% solution. Data for 0, 10, 60, and 120 s of simulation are presented as Panels A–D, respectively. For each time point, the bottom panels depict the distributions of the analytes (left‐hand concentration axis) together with those of the chiral selector and NH_3_ (right‐hand concentration axis). The center panels present the profiles of formic acid (left axis) as well as those of sodium and acetic acid (right axis). The top panels depict the distributions of pH and conductivity. Upon voltage application, anions and cations are migrating toward the anode (left‐hand side) and the cathode (right‐hand side), respectively. Current flow produces an anionically migrating hybrid boundary of CD that comprises a composite of a steady‐state and a continuously broadening part (type of electrophoretic boundary described by Gebauer and Boček [39]). The changing shape is well seen by comparing the HS‐γ‐CD concentration profiles after 10 and 60 s of current flow (bottom graphs in Panels B and C). Two migrating system peaks that originate at the sample‐buffer interfaces are seen in most profiles of the 10 s data of Figure 2B, in which they are marked with asterisks. Chloride is predicted to rapidly leave the sample compartment (bottom panel of Figure 2B), whereas acetic acid is shifting slowly toward the anode only (compare acetic acid data after 10, 60, and 120 s presented in the center panels of Figure 2B–D, respectively). During the 120 s of application of a constant 400 V at a 50 µm ID capillary, the current is predicted to continuously increase from 16.58 to 42.38 µA (inset in the top panel of Figure 2D).

**FIGURE 3:**
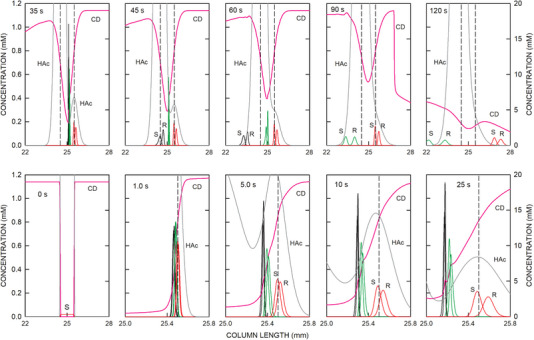
Distributions of the stereoisomers of ketamine (black line graphs), norketamine (green line graphs), and hydroxynorketamine (red line graphs) with the *y*‐axis on the left together with the profiles of HS‐γ‐CD (CD, pink line) and acetic acid (HAc, dark gray line) with the *y*‐axis on the right of the arrangement of Figure 2 within and around the sample compartment after 0, 1.0, 5.0, 10, 25, 35, 45, 60, 90, and 120 s after voltage application. The vertical broken lines demarcate the boundaries of the sampling compartment. S and R refer to stereoisomers of the analytes. The cathode is on the right.

The dynamics of the analytes are presented on an expanded *y*‐axis scale in the graphs of Figure 3. Upon application of voltage, the enantiomers of ketamine, norketamine, and hydroxynorketamine migrate toward the cathode and HS‐γ‐CD in the opposite direction by penetrating into the sample compartment. Complexation is fast and commences when HS‐γ‐CD and analytes meet. Within 1 s of current flow, analytes are predicted to be focused within the CD gradient at the cathodic end of the sample compartment (1 s time point of Figure 3). This gradient remains stationary but broadens with time (compare 1 s data with those of 5, 10, and 25 s presented in the bottom row of Figure 3). As a function of time, its span becomes reduced at the lower side due to diffusion. This is documented with the 35–90 s data in the top row of Figure 3.

S‐ and R‐hydroxynorketamine, which are characterized with the lowest complexation constants (Table 1), partly separate, broaden, and remain stationary up to at least 90 s of current flow. These two analytes become focused at the locations where the CD concentration is such that the effective mobility is zero (about 10.50 and 13.16 mM for S‐ and R‐hydroxynorketamine, respectively, Figure 1). The other two pairs of analytes migrate slowly toward the anode. They have higher complexation constants and lower transitions from cathodic to anodic migration (Table 1 and Figure 1). As the span of the CD gradient becomes sufficiently reduced at the lower side, these analytes switch over into the area of the CD gradient on the anodic side, in which they become slowly defocused and migrate in the anodic direction. The ketamine enantiomers are predicted to leave the sample compartment on the anodic side at around 45 s, whereas those of norketamine are between 80 and 90 s (Figure 3). Finally, upon arrival of the anionically migrating hybrid boundary of CD at the cathodic end of the sample compartment (between 90 and 120 s, Figure 3), the stereoisomers of hydroxynorketamine are starting to migrate into the cathodic part of the column (90 and 120 s data of Figure 3). As the CD concentration continuously decreases, the cationic migration velocity increases and becomes that of the free ion after clearance of the selector. This transition is characterized with a decrease in peak height and an increase in peak width (compare 90 and 120 s data of Figure 3). No further separation of the hydroxynorketamine enantiomers is predicted during migration outside the chiral selector (data not shown).

It is important to note that the simulation was performed for a quiescent solution, that is, without any electroosmosis and/or imposed buffer flow. For the given system with equal column dimensions on either side of the sampling compartment and continued power application, the migration direction of the enantiomers of norketamine and ketamine becomes reversed when the CD concentration at their locations reaches a lower level as that of the transition points of the mobility versus HS‐γ‐CD concentration curves depicted in Figure 1. They can thus reenter the sampling compartment and finally migrate into the cathodic column and eventually to the detector at its end (simulation data not shown). This, however, is not the case in a setup with a larger column diameter on the anodic side, which is most often the case in practice. For this situation, analytes migrating into the anodic vial are thereby typically lost for the analysis. In the presence of a residual electroosmotic flow toward the cathode, however, the sample compartment becomes shifted slowly toward the cathode, and analytes in the vicinity of the anodic part might be able to reach the detector as well.

In the presented simulation, the mobilities of the free analytes were assumed to be equal (Table 1). Measurements with the CE‐MS setup revealed that this represents a good assumption, as mobilities of ketamine, norketamine, and 6‐hydroxynorketamine in the absence of complexation with HS‐γ‐CD were determined to be 2.46 × 10^−8^, 2.49 × 10^−8^, and 2.36 × 10^−8^ m^2^/Vs, respectively. Important for enantiomer separation are the differences of complexation constants and/or differences of the mobilities of the complexes, as was shown in previous simulation work [32, 40, 41, 42]. The example presented in Figures 2 and 3 illustrates that stereoisomers of bases with a low complexation constant can be selectively separated from other analytes of similar chemical structures and analyzed at the cathodic column end with partial filling of a negatively charged chiral selector at high concentration. Parameters affecting this separation are further discussed below. The transient focusing of analyte–CD complexes described is comparable to the electrophoretic focusing of ampholytes in a pH gradient, a process that is referred to as isoelectric focusing [43, 44].

### 3.2 The Impacts of Chiral Selector and Sample Zones

**FIGURE 4:**
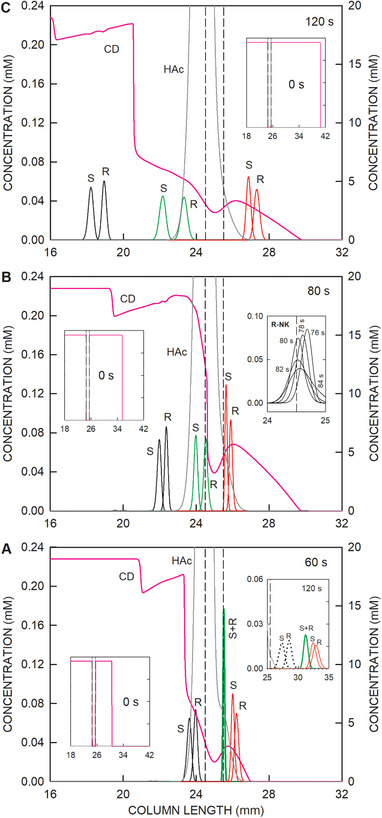
Distributions of the stereoisomers of ketamine (black line graphs), norketamine (green line graphs), and hydroxynorketamine (red line graphs) with the *y*‐axis on the left together with the profiles of HS‐γ‐CD (CD, pink line) and acetic acid (HAc, dark gray line) with the *y*‐axis on the right within and around the sample compartment for (A) a 5 mm HS‐γ‐CD zone and 60 s of voltage application, (B) a 10 mm HS‐γ‐CD zone and 80 s, and (C) a 15 mm HS‐γ‐CD zone and 120 s (as for Figures 2 and 3). S and R refer to stereoisomers of the analytes. All three panels show the initial distributions of HS‐γ‐CD as insets. The second inset in Panel A represents data after 120 s of voltage application with the ketamine profiles as dotted lines as their migration direction became reversed around 60 s of current flow. The second inset in Panel B depicts the behavior of R‐norketamine between 78 and 84 s (2 s interval) of current flow and the thereby associated reversal of migration direction at 80 s. The vertical broken lines demarcate the boundaries of the sampling compartment. The cathode is on the right.

The data presented in Figures 2 and 3 were obtained with a 15 mm zone length of HS‐γ‐CD on the cathodic side of the sampling compartment. Simulation was used to assess at a constant chiral selector concentration the impact of this initial zone length. Zone lengths of 5 mm (Figure 4A), 10 mm (Figure 4B), and 15 mm (Figure 4C) were investigated. The time points chosen for the presented graphs were those at which the anionic migrating analytes within or close to the sampling compartment were reversing their migration direction. In all three cases, hydroxynorketamine stereoisomers are predicted to be on the cathodic side of the sampling compartment. With the shortest CD plug and 60 s of power application, the enantiomers of norketamine are tightly focused at the transition between the sample compartment and cathodic BGE, whereas the enantiomers of ketamine are within the anodic part of the column (Figure 4A). Due to the low amount of chiral selector left at that time and the short remaining exposure of norketamine to the chiral selector, the enantiomers of norketamine cannot be separated upon further application of electric power. This is shown with the 120 s data presented as an inset in Figure 4A. Also presented are the ketamine enantiomers that reversed the migration direction compared to the situation at 60 s and are now also present at the cathodic side of the sampling compartment (dotted line graphs). In reality, these enantiomers could only enter the cathodic column part in the presence of a small flow (e.g., residual electroosmotic flow) toward the cathode and if the instrument features the same capillary ID on the anodic side of the sampling compartment.

When increasing the CD zone length to 10 mm, part of R‐norketamine is recovered only. This is nicely seen with the 76–84 s data presented as an inset in Figure 4B. S‐norketamine and both ketamine enantiomers, however, are within the anolyte and, in the absence of buffer flow, are lost for MS detection at the cathodic column end. Finally, with the 15 mm zone, only the stereoisomers of hydroxynorketamine are predicted to migrate into the cathodic column part and toward the detector (Figures 3 and 4C). The simulation data suggest that the separation of the hydroxynorketamine stereoisomers is independent of the applied zone length of HS‐γ‐CD on the cathodic side of the sampling compartment. During the method development of the CE–MS assay with partial filling of HS‐γ‐CD, similar analyte behavior was observed and led to the decision to focus only on the separation and detection of four hydroxynorketamine stereoisomers and exclude ketamine and norketamine enantiomers from the analysis [17].

**FIGURE 5:**
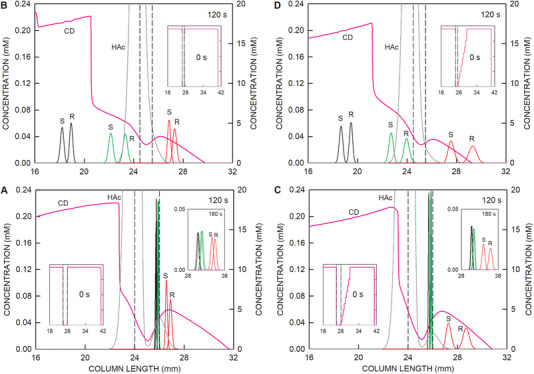
Distributions of the stereoisomers of ketamine (black line graphs), norketamine (green line graphs), and hydroxynorketamine (red line graphs) with the *y*‐axis on the left together with the profiles of HS‐γ‐CD (CD, pink line) and acetic acid (HAc, dark gray line) with the *y*‐axis on the right after 120 s voltage application for a sample compartment length of (A and C) 2 mm and (B and D) 1 mm with (A and B) two sharp sample‐BGE boundaries and (C and D) with a shallow HS‐γ‐CD gradient on the cathodic side of the sample and a sharp boundary on the anodic side. Analyte concentrations applied for the simulations of Panels A and C (10 µM of each stereoisomer) were half of those used for Panels B and D (20 µM of each stereoisomer) such that the sampled amounts were identical in all four simulations. All panels show the initial distributions of HS‐γ‐CD as insets. The second inset in Panels A and C represent 180 s data of the analytes. S and R refer to stereoisomers of the analytes. The vertical broken lines demarcate the boundaries of the sampling compartment. The cathode is on the right.

The sample zone length and sample composition are other parameters that have an impact on analyte selection. The data presented in Figure 5A were simulated with an initial sample zone length of 2 mm, which is twice as long compared to that used for all other simulations, and half of the analyte concentrations such that the total amount sampled was identical. The zone length of the chiral selector was kept at 15 mm as for the data of Figures 2 and 3. For that case, simulation revealed that all six analytes are first focused and then migrate cationically (Figure 5A). This is distinctly different compared to the case with the application of a 1 mm sample (Figure 5B). With the longer sample zone, however, the enantiomers of ketamine and norketamine cannot be resolved because they are not sufficiently exposed to the chiral selector (180 s data presented as an inset in Figure 5A). Similar data for ketamine and norketamine were obtained with an initial stepwise increase of the HS‐γ‐CD concentration on the cathodic side of the sample that resulted in a shallower HS‐γ‐CD concentration gradient (Figure 5C,D). With this HS‐γ‐CD distribution, a much higher resolution of the hydroxynorketamine stereoisomers is predicted compared to the situation of Figure 5A,B. This further illustrates that the separation of weakly complexed analytes occurs within the HS‐γ‐CD gradient. The simulation data suggest that elongation of the initial sample zone length is not advantageous, whereas a shallower HS‐γ‐CD gradient on the cathodic side of the sample provides increased resolution of hydroxynorketamine stereoisomers.

**FIGURE 6:**
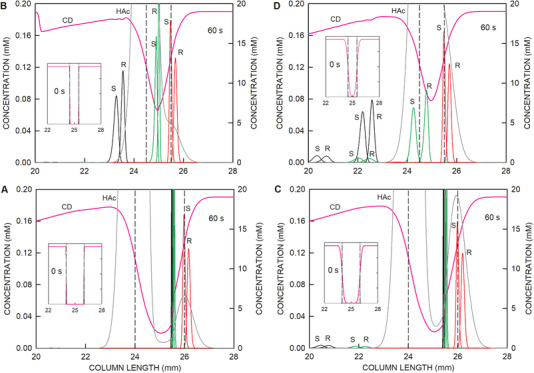
Distributions of the stereoisomers of ketamine (black line graphs), norketamine (green line graphs), and hydroxynorketamine (red line graphs) with the *y*‐axis on the left together with the profiles of HS‐γ‐CD (CD, pink line) and acetic acid (HAc, dark gray line) with the *y*‐axis on the right after 60 s voltage application for a sample compartment length of (A and C) 2 mm and (B and D) 1 mm with sample‐BGE boundary widths of (A and B) 0.05 mm and (C and D) 0.5 mm. Analyte concentrations applied for the simulations of Panels A and C (10 µM of each stereoisomer) were half of those used for Panels B and D (20 µM of each stereoisomer) such that the sampled amounts were identical in all four simulations. All panels show the initial distributions of HS‐γ‐CD as insets. S and R refer to stereoisomers of the analytes. The vertical broken lines demarcate the boundaries of the sampling compartment. The cathode is on the right.

In another effort, simulations were generated with broader sample‐BGE boundaries on both sides of the initial sample zone (Figure 6). Simulation revealed that strongly complexed analytes undergo partial peak splitting when the sample‐BGE boundary width is increased (Figure 6C,D, boundary width of 0.50 mm), a phenomenon that is not predicted with sharp boundaries (Figure 6A,B, boundary width of 0.05 mm). Part of the ketamine and norketamine enantiomers migrates anionically right from the beginning, separates, and produces small peaks (Figure 6C,D). This effect is more pronounced with the application of a 1 mm sample (Figure 6D) compared to the case with a 2 mm sample (Figure 6C). Large parts of the ketamine and norketamine stereoisomers become first focused within the HS‐γ‐CD gradient on the cathodic side and eventually become released into the anodic side, as was described with the data presented in Figure 3. Peak splitting is not predicted for the weakly complexed hydroxynorketamine stereoisomers. Thus, the described assay appears to be ideal for analysis of these compounds, particularly with a shallower HS‐γ‐CD gradient on the cathodic side of the sample.

**FIGURE 7:**
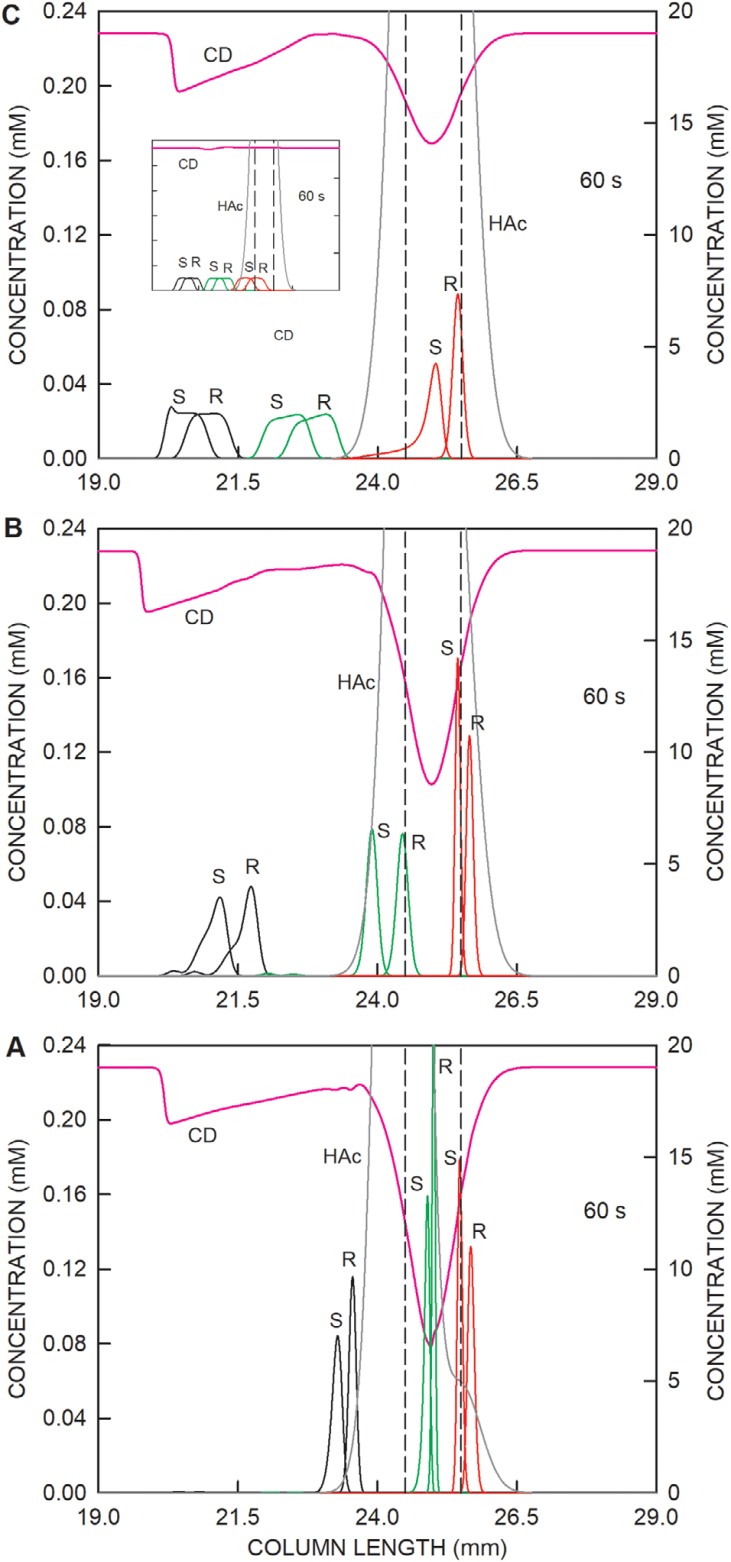
Distributions of the stereoisomers of ketamine (black line graphs), norketamine (green line graphs), and hydroxynorketamine (red line graphs) with the *y*‐axis on the left together with the profiles of HS‐γ‐CD (CD, pink line) and acetic acid (HAc, dark gray line) with the *y*‐axis on the right within and around the sample compartment after 60 s of voltage application for the sample prepared (A) without BGE (as for Figure 2), (B) in 10‐fold diluted BGE with its 1.9 mM HS‐γ‐CD, and (C) in 2‐fold diluted BGE with 9.5 mM of HS‐γ‐CD. The inset in Panel C depicts data obtained with the sample prepared in BGE (19 mM HS‐γ‐CD). The vertical broken lines demarcate the boundaries of the sampling compartment. The cathode is on the right. S and R refer to stereoisomers of the analytes.

Finally, the composition of the sample was investigated. Data obtained for 60 s of simulation of four different sample matrices are presented in Figure 7. Graphs obtained for a sample consisting of the analytes in 83 mM acetic acid (as for Figures 2 and 3) are depicted in Panel A of Figure 7, whereas those containing 10‐ and 2‐fold diluted BGE with ammonium formate, formic acid, HS‐γ‐CD, and sodium together with 83 mM acetic acid are shown in Panels B and C of Figure 7, respectively. In comparison to a sample without BGE, the addition of 10‐fold diluted BGE with its 1.9 mM HS‐γ‐CD revealed data that exhibit a faster removal of the enantiomers of ketamine and norketamine into the anolyte. The behavior of the hydroxynorketamine stereoisomers, however, is not affected by the additional sample matrix (Figure 7A,B). With two‐fold diluted BGE with its 9.5 mM HS‐γ‐CD in the sample, R‐hydroxynorketamine remains focused at the cathodic end of the sample compartment and then migrates toward the detector, whereas part of S‐hydroxynorketamine migrates into the anodic compartment and is lost for the analysis (Figure 7C). With an even higher BGE content in the sample, which is associated with a further increase of the CD concentration, this situation becomes changed such that both S‐ and R‐hydroxynorketamine migrate anionically. This is illustrated for the case with undiluted BGE (19 mM HS‐γ‐CD) in the sample (inset in Figure 7C). Thus, the presence of HS‐γ‐CD in the sample may influence analyte migration such that its detection on the cathodic side is hindered. In all cases, simulation data reveal that salt present in the sample is quickly removed from the sampling compartment. Anions are migrating into the anolyte, whereas cations are migrating into the catholyte. This is, for example, shown with the removal of chloride (bottom panel in Figure 2B).

### 3.3 Change of the Chiral Selector Concentration in the Catholyte

**FIGURE 8:**
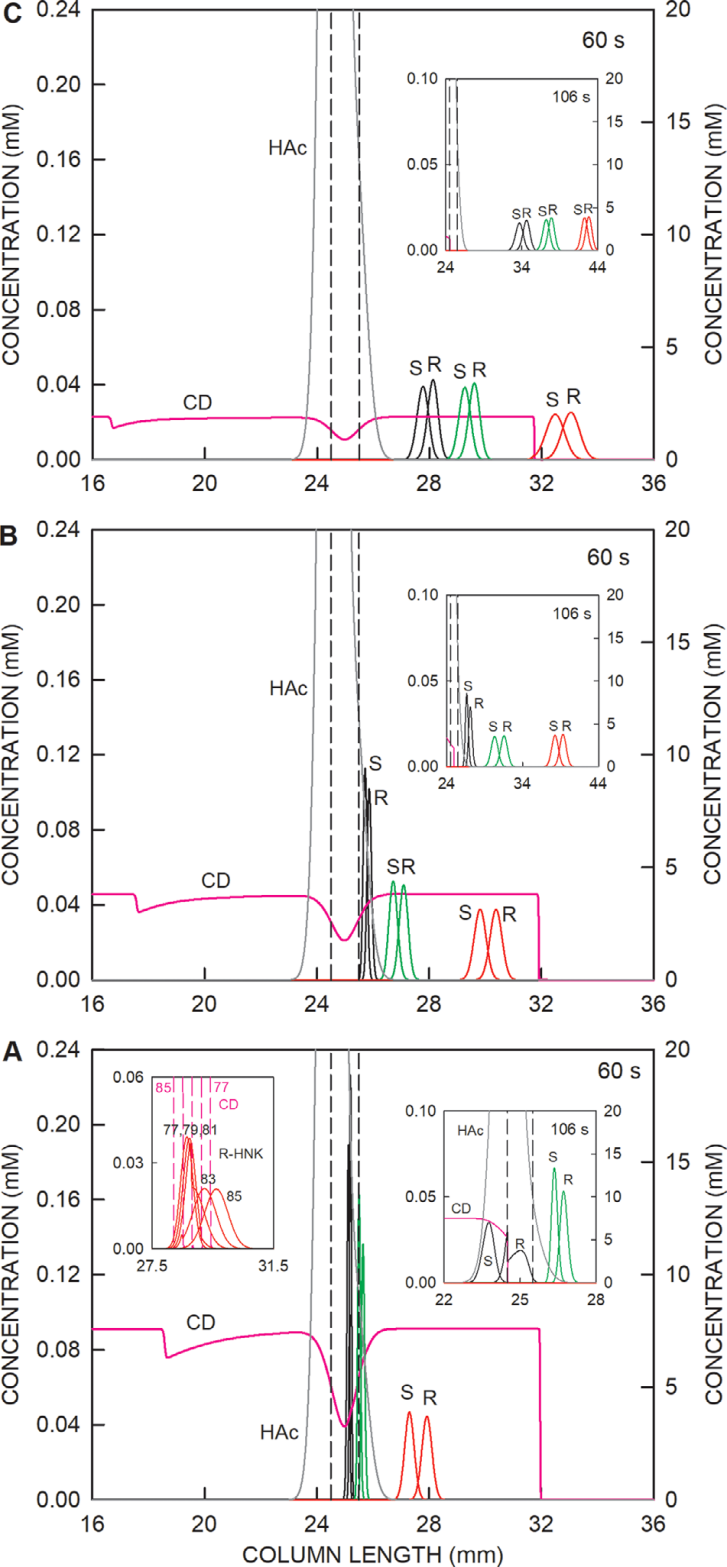
Distributions of the stereoisomers of ketamine (black line graphs), norketamine (green line graphs), and hydroxynorketamine (red line graphs) with the *y*‐axis on the left together with the profiles of HS‐γ‐CD (CD, pink line) and acetic acid (HAc, dark gray line) with the *y*‐axis on the right within and around the sample compartment for (A) 7.6 mM HS‐γ‐CD and 98.8 mM NaOH, (B) 3.8 mM HS‐γ‐CD and 49.4 mM NaOH, and (C) 1.9 mM HS‐γ‐CD and 24.7 mM NaOH, 60 s of voltage application, and otherwise identical conditions as for Figure 2. The insets on the right side of each panel depict data obtained after 106 s of voltage application. The left inset in Panel A shows the peak shape change of R‐hydroxynorketamine that occurs between 77 and 85 s (data presented at 2 s intervals presented with red solid lines) when the analyte is leaving the HS‐γ‐CD zone (positions of the HS‐γ‐CD boundaries marked with pink broken lines). For details, refer to text. The vertical broken lines demarcate the boundaries of the sampling compartment. The cathode is on the right. S and R refer to stereoisomers of the analytes.

In the simulations presented thus far, a 19 mM HS‐γ‐CD concentration was used. This corresponds to the 5% solution of the CE‐MS assay of Sandbaumhüter et al. [17] and provides an environment in which all studied bases are migrating anionically (Figure 1 and inset in Figure 7C). The use of lower concentrations, particularly those that enable a cationic migration of hydroxynorketamine, was investigated. Simulation data obtained with 7.6 mM HS‐γ‐CD (2% solution of chiral selector with 98.8 mM NaOH), 3.8 mM HS‐γ‐CD (1% solution with 49.4 mM NaOH), and 1.9 mM HS‐γ‐CD (0.5% solution with 24.7 mM NaOH) and otherwise identical conditions as for the case with 19 mM HS‐γ‐CD of Figures 2 and 3 are presented in Panels A–C of Figure 8, respectively. For the three CD concentrations of Figure 8, simulation predicts an anionically migrating HS‐γ‐CD boundary with a steady‐state shape. This is distinctly different from the hybrid boundary in the case of 19 mM HS‐γ‐CD (Figure 2).

With 7.6 mM HS‐γ‐CD, the 60 s profiles reveal that the stereoisomers of hydroxynorketamine are indeed predicted to migrate cationically and to separate while exposed to the chiral selector (Figure 8A). At this time, the enantiomers of norketamine are still focused but begin to migrate into the cathodic column part, whereas the enantiomers of ketamine are tightly focused within the HS‐γ‐CD gradient and do not migrate. Prolonged application of voltage results in a slow anionic release of S‐ketamine into the anolyte. The same is true for a part of R‐ketamine. At 106 s of power application, the anionically migrating CD boundary reaches the cathodic end of the sampling compartment (inset in Figure 8A). From that time onward, the part of R‐ketamine in the sampling compartment migrates cationically in the absence of the chiral selector such that it remains available for analysis at the cathodic column end. The use of 3.8 mM HS‐γ‐CD predicts the cationic migration of all investigated bases (Figure 8B). The same is true for 1.9 mM HS‐γ‐CD (Figure 8C). These data also reveal that stereoisomer separation becomes slower with reduction of the chiral selector concentration. Longer zones of the BGE with the selector are required for full separation of the stereoisomers. Those findings are in agreement with the observations during method development described previously [17].

The transition of analytes from the CD‐containing BGE into CD‐free solution is characterized with a strong migration rate increase (increase in effective mobility to the mobility of the free ions). This process is accompanied with a strong decrease in peak height and an increase in peak width as the front boundary becomes unexposed to the chiral selector earlier than the back boundary. For the case with 7.6 mM HS‐γ‐CD this effect is illustrated with the 77–85 s data of R‐hydroxynorketamine (red lines) and HS‐γ‐CD (dashed pink lines) at a two‐second interval presented in the left insert of Figure 8A. Note that R‐hydroxynorketamine is migrating toward the cathode, whereas HS‐γ‐CD in the opposite direction. No further separation of the enantiomers is predicted during migration outside of the section with the chiral selector as the migration rates of both enantiomers become equal. The peak shape change is comparable to that predicted for configurations with neutral chiral selectors [34]. Simulation is the only means for the visualization of this electrophoretic transition.

## 4 Concluding Remarks

Simulations revealed the dynamics of weak bases as analytes in an enantioselective CE–MS system with HS‐γ‐CD as a chiral selector applied in partial filling mode. Parameters that affect the chiral separation of ketamine, norketamine, and hydroxynorketamine and the detection possibility of the analytes at the cathodic side of the electrophoretic column were thereby identified. Chiral separation and migration direction are dependent on the CD concentration. With a CD concentration below the characteristic concentration at which the migration direction changes from cationic to anionic transport, enantiomers migrate toward the cathode, gradually separate when effective mobilities are sufficiently different, and experience a dilution at the transition into the CD‐free part of the column. For the case with a high HS‐γ‐CD concentration at which the analytes migrate anionically, as was employed in the CE–MS assay reported previously [17], the situation is much different. Enantiomer separation and migration toward the cathode can only be achieved for weakly complexed analytes such as hydroxynorketamine and provided that the sample is applied without or with a small amount of the chiral selector only. Upon current application, analytes become quickly focused and separated in the thereby formed gradient at the cathodic end of the sample compartment. This gradient ranges from a low to a high HS‐γ‐CD concentration, broadens with time, remains stationary, and gradually reduces its span at the lower side due to diffusion such that analytes with high affinity to the anionic selector become slowly released onto the other side of the focusing gradient where anionic migration and defocusing occur concomitantly. In the absence of buffer flow toward the cathode, these analytes are lost for detection on the cathodic column side. The analytes that remain focused until the HS‐γ‐CD concentration boundary arrives at the cathodic end of the sample compartment gradually become released into the cathodic part and migrate toward the detector. This behavior was found to be dependent on the length of the CD zone and the initial sample zone length. The data presented also illustrate the possibility that only one of the enantiomers of an analyte migrates toward the detector whereas the other is lost for the analysis or that both enantiomers migrate toward the cathode but do not separate. Simulation data provided insight into the nature of all migrating and stationary boundaries present in the electrophoretic system in a straightforward way and thereby permitted the elucidation of the separation principle that led to the selective analysis of urinary hydroxynorketamine stereoisomers in the CE–MS assay with partial filling of a high concentration of HS‐γ‐CD [17]. For the assessed system, analyte behavior is shown to be dependent on complexation with HS‐γ‐CD, sample zone length, and sample composition, whereas enantiomeric analyte separation for detection on the cathodic side is dependent on the slope of the HS‐γ‐CD focusing gradient. Selective analysis of the stereoisomers of weakly complexed bases can be achieved in a rather short column and thus could be implemented on a microchip setup. The presented work illustrates that dynamic simulation is an indispensable tool to investigate electrophoretic processes of complex systems. Computer modeling is essential to describe the focusing of the analytes in the HS‐γ‐CD gradient and to elucidate the processes that are associated with the slowly changing HS‐γ‐CD distributions in the sample compartment. The understanding of other phenomena, including the migration direction at a given HS‐γ‐CD concentration, can also be assessed without modeling [19].

## Conflicts of Interest

The authors declare no conflicts of interest.

## Data Availability

The data that support the findings of this study are available from the corresponding author upon reasonable request.
